# Development of a quantitative food frequency questionnaire for Brazilian patients with type 2 diabetes

**DOI:** 10.1186/1471-2458-13-740

**Published:** 2013-08-09

**Authors:** Roberta Aguiar Sarmento, Bárbara Pelicioli Riboldi, Ticiana da Costa Rodrigues, Mirela Jobim de Azevedo, Jussara Carnevale de Almeida

**Affiliations:** 1 Endocrinology Division, Hospital de Clínicas de Porto Alegre, Universidade Federal do Rio Grande do Sul, Rio Grande do Sul, Brazil; 2 Nutrition Graduate Program, Faculdade de Medicina, Universidade Federal do Rio Grande do Sul, Porto Alegre, Rio Grande do Sul, Brazil; 3 Department of Internal Medicine, Faculdade de Medicina, Universidade Federal do Rio Grande do Sul, Porto Alegre, Rio Grande do Sul, Brazil

**Keywords:** Food frequency questionnaire, Type 2 diabetes mellitus, Food record, Epidemiologic methods

## Abstract

**Background:**

To investigate the association between dietary components and development of chronic diabetic complications, the dietary evaluation should include a long period, months or years. The present manuscript aims to develop a quantitative food frequency questionnaire (FFQ) and a portfolio with food photos to assess the usual intake pattern of Brazilian patients with type 2 diabetes to be used in future studies.

**Methods:**

Dietary data using 3-day weighed diet records (WDR) from 188 outpatients with type 2 diabetes were used to construct the list of usually consumed foods. Foods were initially clustered into eight groups: “cereals, tubers, roots, and derivatives”; “vegetables and legumes”; “fruits”; “beans”; “meat and eggs”; “milk and dairy products”; “oils and fats”, and “sugars and sweets”. The frequency of food intake and the relative contribution of each food item to the total energy and nutrient intakes were calculated. Portion sizes were determined according to the 25^th^, 50^th^, 75^th^, and 95^th^ percentiles of intake for each food item.

**Results:**

A total of 62 food items were selected based on the 3-day WDR and another 27 foods or how they are prepared and nine beverages were included after the expert examination. Also, a portfolio with food photos of each included food item and portion sizes was made to assist the patients in identifying the consumed portion.

**Conclusions:**

We developed a practical quantitative FFQ and portfolio with photos of 98 food items covering those most commonly consumed in the past 12 months, to assess the usual diet pattern of patients with type 2 diabetes in Southern Brazil.

## Background

The field of nutritional epidemiology has been developed because of an interest in the concept that aspects of diet may influence the occurrence of human disease [1]. In the case of patients with diabetes, dietary advice and assessment of compliance with these recommendations are important for achieving metabolic goals, especially glycemic control [2].

There are several methods for the assessment of food and nutrient consumption as well as energy intake, including 24-hour recall, food records, food frequency questionnaire (FFQ), and biomarkers [3]. To investigate the association between dietary components and development of chronic diabetic complications, the dietary evaluation should include a long period, months or years, as is the case of FFQ. To date, four FFQs involving patients with diabetes have been validated and published in specific populations: Australian [4], Japanese [5], Malian [6], and Korean [7]; however, none was made for the Brazilian population. In fact, the FFQ should represent regional habits and the accuracy of such data needs to take this into account [8].

In drawing up an FFQ, careful attention must be given to the choice of foods, the clearness of the questions, and the format of the frequency response section. In addition, the choice of foods, especially if the FFQ is constructed to also include quantitative or semi-quantitative dietary evaluation, should be based on an accurate dietary tool [9]. In this way, the present manuscript aims to create an FFQ and a portfolio with food photos to assess the usual intake pattern of Brazilian patients with diabetes to be used in future studies.

## Methods

### Study population

Patients were identified belonged to the Group of Nutrition in Endocrinology (GNE), a cohort of outpatients with type 2 diabetes in southern Brazil [10]. The GNE study was designed to evaluate possible associations of dietary factors with chronic complications of diabetes. From a previously constructed database of patients with type 2 diabetes [11] data from consecutive registered patients who reported a plausible ratio of protein intake estimated from the 3-day weighed diet records (WDR) to protein intake from urinary nitrogen [12] were selected. The acceptable ratio between the two protein intake estimates ranged from 0.79 to 1.26 [12]. An equal seasonal distribution (1:1:1:1 spring, summer, autumn, and winter) and the same gender proportion (1:1 males and females) between each season were also considered inclusion criteria. Therefore, records from 188 patients with type 2 diabetes were analyzed.

This study was conducted according to the guidelines laid down in the Declaration of Helsinki and all procedures involving patients were approved by the Hospital Ethics Committee. Written informed consent was obtained from all patients.

### The new instrument: food frequency questionnaire

The most frequently consumed foods and their respective portion sizes were extracted from 3-day WDR (two nonconsecutive weekdays and one-weekend day) to create the FFQ and the food portfolio photo. All registered foods and preparation methods were listed and clustered into eight groups as proposed by the Food Guide for the Brazilian Population [13]: “cereals, tubers, roots, and derivatives”; “vegetables and legumes”; “fruits”; “beans”; “meat and eggs”; “milk and dairy products”; “oils and fats” and “sugars and sweets”. The caloric and non caloric beverages were added into a new group, according to the WDR description (“beverages group”).

#### Data analyses

A food item was classified according to its relative contribution, at least 80%, for daily energy or intake of a selected relevant nutrient (K nutrient) in its respective food group. The relative contribution was calculated by the equation proposed by Block et al. [14] [% K nutrient contribution by food = (amount of the K nutrient provided by food × 100) / amount of the K nutrient provided by all foods]. The most relevant nutrients in each food group were selected considering their influence on glucose metabolism [15-18] and/or diabetic complications [15,19-22] and are described in Table 1. Information about the nutritional composition of each food and regional ingredients used in their preparation was based on NutriBase Clinical® software (1986-2013 CyberSoft, Inc. an Arizona corporation). This software used the USDA Nutrient Database for Standard Reference [23]. Nutrient data on frequently consumed foods were complemented if necessary with data obtained from local manufacturers of specific industrialized foods.

**Table 1:** **The most relevant nutrients in each food group considering their influence on glucose metabolism and**/**or diabetic complications**

**Food group**	**Nutrients**
Cereals, tubers, roots and derivatives	Carbohydrate
Vegetables and legumes	Fiber, iron, calcium, and potassium
Fruits	Carbohydrate, fiber, and potassium
Beans	Protein, fibers, and iron
Meat and eggs	Protein, lipids, and iron
Milk and dairy products	Protein, lipids, and calcium
Oils and fats	Lipids
Sugars and sweets	Carbohydrate

**Figure 1:**
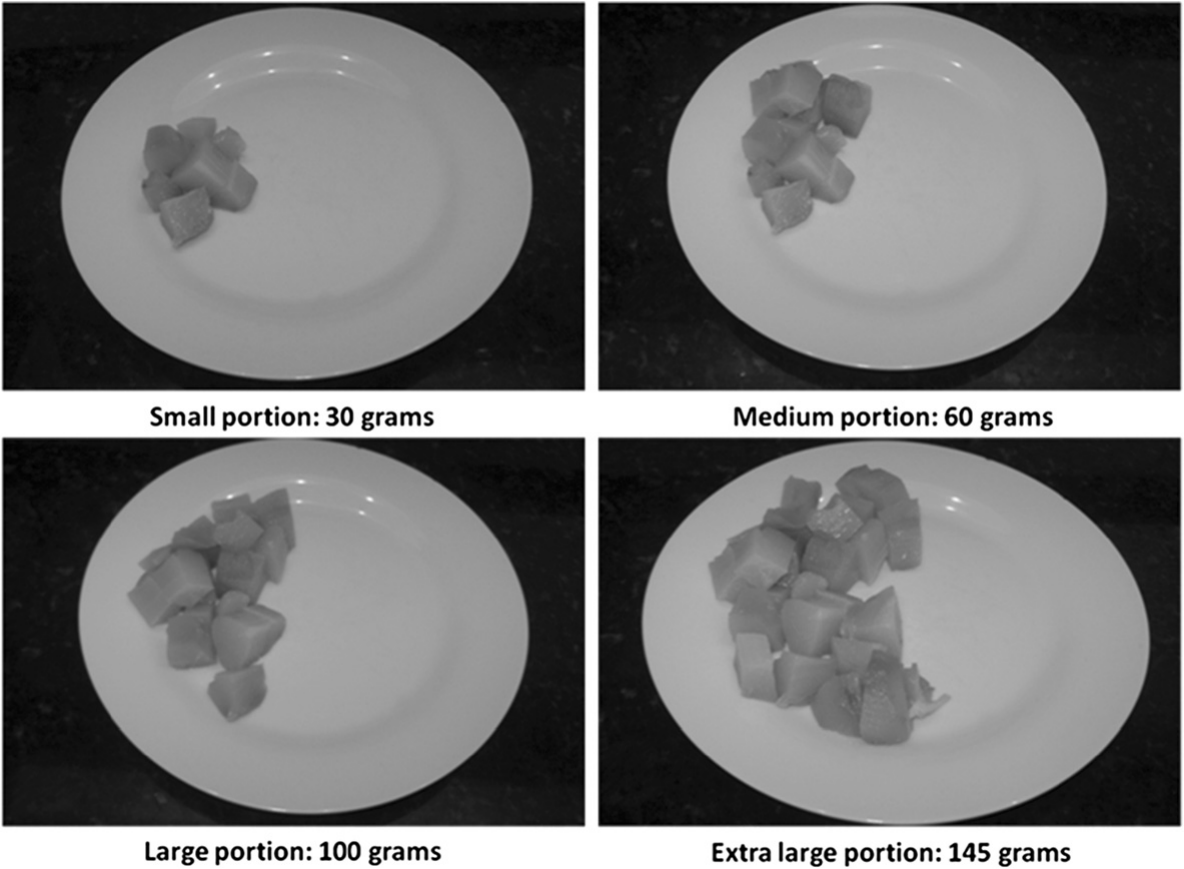
Illustration of four portions of the same food (chayote cooked) photographed and included on the food portfolio.

The size of servings of each food item was classified according to its respective weight distribution as registered in the WDR: small = 25^th^ percentile, medium = 50^th^ percentile, large = 75^th^ percentile, and extra large = 95^th^ percentile [24]. Figure 1 shows an example of food portions as illustrated in the food portfolio photo. The amount of each portion in grams or milliliters was transformed into household measures using the Table for Assessment of Food Intake in Household Measures [25]. The FFQ also included open questions about the frequency of food consumption and an option to include new foods according to personal eating habits. The frequency was described as the number of times the food was consumed and also if the intake occurred daily, weekly, monthly, or yearly.

In order to obtain an expert examination, the constructed FFQ was submitted to health researchers used to dealing with diabetes care: endocrinologists, nutritionists, and researchers from the GNE [10]. After the experts’ meeting, changes were made in the food list and definition of portion sizes. Regional dishes and seasonal foods were also included according to suggestions.

### Portfolio with food photos

The construction of the portfolio with food photos was based on the methodology suggested by Monteiro et al. [26]. Digital photographs were taken of each portion of food from the FFQ and organized in the order in which they were mentioned, considering the four portion sizes and food groups (Figure 1). A numerical legend was also created to explain details about the each portion (amount in grams or milliliters) and keep patients blinded to serving sizes. The food portions were determined with an analytical scale (Marte ®, from 0.01 to 2000 g) and measuring cup (50-250 mL; Marinex, Brazil). The solid foods were arranged in the same plate meal size to perform the pictures, in order to help the patients acquire a perspective of size.

## Results

The main features of 188 patients with type 2 diabetes were: 61.1 ± 10.1 years of age (range 34-80 years), males 50.0%, 12 years (6-18 years) of diabetes duration, BMI of 28.8 ± 4.3 kg/m^2^; HbA1c of 7.5 ± 1.4%, 42.5% from lower middle class, and 84.4% self-identified as whites. The patients performed 3-day WDR, totaling 564 WDR during all seasons: 25% (n = 141) in winter, 25% (n = 141) in spring, 25% (n = 141) in summer, and 25% (n = 141) in autumn.

Initially, a list of 177 different food items was compiled based on data from the WDR and the number of food items in each food cluster was as follows: “cereals, tubers, roots, and derivatives” - 39 food items; “vegetables and legumes” - 34 food items; “fruits”- 22 food items; “beans” - 5 food items; “meat and eggs” - 27 food items; “milk and dairy products” - 14 food items; “oils and fats” - 7 food items; “sugars and sweets”- 16 food items; “beverages” - 13 food items. Subsequently, only 62 food types were included in the FFQ, considering the 80% cutoff contribution in its respective food group. The reported frequency of each included food item with respective relevant nutrient is shown in Table 2. The most frequently consumed foods by patients with diabetes included white rice (94.1%), papaya (87.2%), beans (78.2%), French or Vienna bread (75.5%), banana (71.8%), and tomato (71.3%). Furthermore, another four food items (lettuce, beef, chicken, and margarine) were reported by more than 50% of this patient sample. After expert examination, 21 regional foods (fruits, vegetables, sweets, and fats), six different types of food preparations, and nine beverages were included in the food list.

**Table 2:** Food list from food frequency questionnaire for diabetes: registered consumption frequency of 188 patients with type 2 diabetes and nutrient contribution

			Nutrient contribution*							
**Foods**	**Subjects consuming this food**		**Calories**	**Carbohydrate**	**Protein**	**Lipid**	**Fiber**	**Iron**	**Calcium**	**Potassium**
	**n**	**%**								
**Cereals, tubers, roots and derivatives**										
White rice	177	94.1	yes	yes	yes	no	yes	yes	no	yes
French or Vienna bread	142	75.5	yes	yes	yes	yes	yes	yes	yes	yes
Spaghetti pasta	76	40.4	yes	yes	yes	no	yes	yes	no	yes
Wheat cracker	82	43.6	yes	yes	yes	yes	yes	yes	yes	yes
Whole bread	78	41.4	yes	yes	yes	yes	yes	yes	yes	yes
Cassava, boiled	41	21.8	yes	yes	no	no	yes	no	no	yes
Cake	35	18.6	yes	yes	no	yes	yes	yes	no	yes
Maize porridge	23	12.2	yes	yes	no	no	yes	yes	no	yes
Potato, boiled/baked	82	43.6	yes	yes	no	no	yes	no	no	yes
Homemade bread	24	12.7	yes	yes	no	no	no	yes	no	no
White bread	35	18.6	yes	yes	no	no	no	yes	yes	no
Milk cracker	27	14.3	yes	yes	no	yes	no	yes	yes	no
**Vegetables and legumes**										
Carrot	77	40.9	no	no	no	no	yes	no	yes	yes
Cabbage	56	29.7	no	no	no	no	yes	yes	yes	yes
Tomato	134	71.2	no	no	no	no	yes	no	no	yes
Chayote	34	18.0	no	no	no	no	yes	no	no	yes
Lettuce	112	59.5	no	no	no	no	no	yes	yes	yes
Kale	37	19.6	no	yes	no	no	no	yes	yes	yes
Broccoli	22	11.7	no	no	no	no	no	no	no	yes
Pumpkin	17	9.0	no	no	no	no	no	no	no	yes
Beet	20	10.6	no	no	no	no	no	no	no	yes
**Fruits**										
Banana	135	71.8	yes	no	no	no	yes	yes	no	yes
Apple	92	48.9	yes	yes	no	no	yes	no	no	yes
Orange	58	30.8	yes	yes	no	no	yes	no	yes	yes
Tangerine	51	27.1	yes	yes	no	no	yes	no	yes	yes
Papaya	164	87.2	yes	yes	no	no	yes	no	yes	yes
Mango	16	8.5	no	no	yes	no	no	no	no	no
Pear	19	10.1	no	no	no	no	yes	no	no	no
**Beans**										
Beans (all types)	147	78.1	yes	yes	yes	no	yes	yes	yes	yes
Lentil	16	8.5	yes	yes	no	no	yes	yes	no	yes
**Meat and eggs**										
Beef, boiled/baked	122	64.8	yes	no	yes	yes	no	yes	yes	yes
Chicken, boiled/baked	123	65.4	yes	no	yes	yes	no	yes	yes	yes
Ground beef	63	33.5	yes	no	yes	yes	no	yes	yes	yes
Beef steak	64	34.0	yes	no	yes	yes	no	yes	no	yes
Luncheon/bologna	43	22.8	yes	no	yes	yes	no	yes	no	yes
Fish, boiled/baked	21	11.1	yes	no	yes	yes	no	no	no	yes
Pork	28	14.8	yes	no	yes	yes	no	yes	no	yes
Fish, fried	9	4.7	yes	no	no	yes	no	no	no	yes
Chicken, fried	14	7.4	yes	no	yes	no	no	no	no	yes
Frankfurter wiener, hot dog	15	7.9	no	no	no	yes	no	no	no	no
Mortadella	35	18.6	no	no	no	yes	no	no	no	no
Salami	23	12.2	no	no	no	yes	no	no	no	no
Beef, fried	8	4.2	no	no	no	yes	no	no	no	no
Egg, boiled/fried	22	11.7	no	no	no	yes	no	no	no	no
Beef liver	6	3.1	no	no	no	no	no	yes	no	no
Ham	48	25.5	no	no	no	no	no	no	no	yes
**Milk and dairy products**										
Muenster cheese	76	40.4	yes	no	yes	yes	no	no	yes	no
Milk, fluid, 3.25% fat	73	38.8	yes	yes	yes	yes	no	no	yes	yes
Milk, fluid, nonfat	78	41.4	yes	yes	yes	no	no	no	yes	yes
Goat cheese, soft type	32	17.0	no	no	no	yes	no	no	yes	no
Muenster cheese	12	6.3	no	no	no	yes	no	no	yes	no
Milk, fluid, 2% fat	19	10.1	no	no	no	no	no	no	yes	yes
Yogurt, plain	10	5.3	no	no	no	no	no	no	yes	no
Milk type C	10	5.3	no	no	no	no	no	no	yes	no
Yogurt, plan, skim	8	4.2	no	no	no	no	no	no	yes	no
Milk, dry, whole	10	5.3	no	no	no	no	no	no	yes	no
Yogurt, fruit	6	3.1	no	no	no	no	no	no	yes	no
American cheese	11	5.8	no	no	no	no	no	no	yes	no
**Oils and fats**										
Margarine	101	53.7	yes	no	no	yes	no	no	no	no
Goose pate	23	12.3	no	no	no	yes	no	no	no	no
Mayonnaise	19	10.1	no	no	no	yes	no	no	no	no
**Sugars and sweets**										
Flan and/or pudding diet	8	4.2	no	no	no	no	no	no	yes	no

*Nutrient contribution defined as contribution of at least 80% of total energy or relevant nutrient intake in the respective food group.

The final version of the FFQ consisted of 98 food items and beverages distributed into nine groups: eight food groups and one of beverages. The preparation options (fried, boiled, cooked or roasted) were considered in food items of the “cereals, tubers, roots, and derivatives” and “meats and eggs” groups. The FFQ is shown in Additional file 1. All included food items contributed 95% of the total energy and nutrient intake as follows: total energy (94.2%), protein (96.8%), carbohydrate (92.8%), fat (94.6%), fiber (90.3%), iron (93.4%), calcium (95.3%), and potassium (92.2%). The portions of each food in grams or milliliters and its respective number of portions in household measures are shown in Table 3.

**Table 3:** Final food list in the food frequency questionnaire: portions in grams or milliliters and household measures

**Food group**	**Small (25**^**th**^**)**		**Medium (50**^**th**^**)**		**Large (75**^**th**^**)**		**Extra large (95**^**th**^**)**	
**Cereals, tubers, roots, and derivatives**								
White rice	2 full tablespoon	50 g	4 full tablespoon	100 g	5 full tablespoon	125 g	8 full tablespoon	200 g
Spaghetti pasta	3 full tablespoon	75 g	4 full tablespoon	100 g	1 paten	200 g	1 full paten	320 g
Cassava, boiled/fried	2 pieces	60 g	3 pieces	90 g	4 pieces	120 g	6 pieces	240 g
Potato, boiled/baked/fried	2 full tablespoon	60 g	3 full tablespoon	90 g	4 full tablespoon	120 g	6 full tablespoon	180 g
Maize porridge, boiled/fried	1 serving spoon	60 g	2 full tablespoon	90 g	3 full tablespoon	150 g	1 paten	325 g
French or Vienna bread	½ unit	25 g	1 unit	50 g	1 and ½ unit	75 g	2 units	100 g
White bread	1 slice	25 g	2 slice	50 g	2 and ½ slices	62.5 g	3 and ½ slices	87.5 g
Whole bread	½ slice	15 g	1 slice	30 g	2 slices	60 g	3 slices	90 g
Homemade bread	2/3 slice	60 g	1 slice	68 g	1 and ½ slice	86 g	2 and ½ slices	145 g
Cake	1 small slice	50 g	1 medium slice	70 g	1 large slice	90 g	2 medium slices	140 g
Wheat cracker	4 units	20 g	6 units	30 g	9 units	45 g	20 units	100 g
Milk cracker	5 units	25 g	8 units	40 g	11 units	55 g	32 units	160 g
**Vegetables and legumes**								
Carrot	2 full tablespoon	24 g	3 full tablespoon	36 g	5 full tablespoon	60 g	10 full tablespoon l	120 g
Tomato	3 small slices	30 g	5 small slices	50 g	7 small slices	70 g	7 medium slices	100 g
Chayote	1 full tablespoon	30 g	2 full tablespoon	60 g	3 and ½ full tablespoon	100 g	5 full tablespoon	145 g
Cabbage	4 full tablespoon	40 g	7 full tablespoon	70 g	10 full tablespoon	100 g	6 full medium skimmer	150 g
Lettuce	1 tagger	20 g	2 taggers	30 g	5 medium leaf	50 g	1 full paten	80 g
Watercress	1 full dessert plate	20 g	2 taggers	30 g	1 full paten	80 g	2 full patens	160 g
Kale, spinach	2 full tablespoon	40 g	3 full tablespoon	60 g	5 full tablespoon	100 g	9 full tablespoon	180 g
Broccoli, cauliflower	1 small bunch	30 g	1 medium bunch	60 g	1 large bunch	100 g	2 medium bunches	130 g
Snap bean	2 level tablespoon	30 g	2 full tablespoon	40 g	5 full tablespoon	100 g	15 full tablespoon l	300 g
Pumpkin	1 medium piece	50 g	2 medium pieces	100 g	2 and ½ medium pieces	125 g	6 medium pieces	300 g
Beet	2 medium slices	30 g	5 medium slices	60 g	8 medium slices	90 g	12 medium slices	140 g
**Fruits**								
Banana	1 small unit	40 g	1 medium unit	70 g	1 large unit	90 g	2 medium units	140 g
Apple, pear	1 small unit	90 g	1 and ½ small unit	135 g	1 medium unit	150 g	1 large unit	230 g
Orange, tangerine	1 small unit	90 g	1 and ½ small unit	135 g	1 large unit	180 g	2 medium units	225 g
Papaya	½ small slice	80 g	1 medium slice	100 g	¼ unit	135 g	½ unit	270 g
Mango	1 small piece	60 g	2 small pieces	120 g	1 medium pieces	140 g	6 small pieces	360 g
Grape	8 units	64 g	14 units	112 g	1 small bunch	170 g	1 medium bunch	350 g
Persimmon	1 small unit	85 g	1 large unit	150 g	2 medium units	220 g	3 small units	255 g
Casaba melon	½ small slice	78 g	1 small slice	125 g	1 medium slice	200 g	1 large slice	300 g
Watermelon	1 small slice	143 g	1 medium slice	200 g	1 large slice	282 g	2 medium slices	350 g
**Beans**								
Beans (all types)	1 small full scoop	65 g	1 level medium scoop	80 g	2 small full scoop	130 g	2 level medium scoop	160 g
Lentil	1 level medium scoop	100 g	1 medium full scoop	160 g	2 level medium scoop	200 g	2 medium full scoop	320 g
**Meat and eggs**								
Beef, boiled/baked/fried	1 small slice	70 g	4 small pieces	80 g	1 large slice	135 g	2 large slices	270 g
Ground beef	2 full tablespoon	50 g	3 full tablespoon	75 g	4 full tablespoon	100 g	8 full tablespoon	200 g
Beef steak	½ small unit	40 g	1 small unit	80 g	1 medium unit	100 g	2 medium units	200 g
Beef liver	½ large unit	75 g	1 small unit	80 g	1 medium unit	100 g	1 large unit	150 g
Chicken thigh, boiled/baked/fried	1 medium piece	60 g	1 large piece	95 g	2 medium pieces	110 g	3 medium pieces	180 g
Chicken breast, boiled/baked/fried	1 medium piece	60 g	1 large piece	95 g	2 medium pieces	110 g	3 medium pieces	180 g
Fish, boiled/baked/fried	½ small piece	60 g	1 small piece	100 g	1 large piece	155 g	2 large pieces	310 g
Pork, boiled/baked/fried	1 small slice	60 g	1 medium slice	90 g	1 large slice	120 g	2 medium slices	180 g
Luncheon/bologna	½ unit	30 g	1 unit	60 g	1 and ½ units	90 g	2 and ½ units	150 g
Frankfurter wiener, hot dog	1 unit	42 g	1 and ½ unit	63 g	2 units	84 g	3 and ½ units	147 g
Mortadella, ham, salami	1 medium slice	15 g	1 large slice	25 g	2 medium slices	30 g	2 large slices	50 g
Egg, boiled/fried	½ unit	25 g	1 unit	50 g	1 and ½ unit	75 g	3 units	150 g
**Milk and dairy products**								
Milk, fluid, 3.25% fat	½ cup	100 ml	¾ cup	150 ml	1 cup	200 ml	1 mug	300 ml
Milk, fluid, 2% fat	½ cup	100 ml	¾ cup	150 ml	1 cup	200 ml	1 mug	300 ml
Milk, fluid, nonfat	¾ cup	150 ml	1 cup	200 ml	1 glass	240 ml	1 and ½ cups	250 ml
Milk, dry	1 full tablespoon	16 g	2 full dessert spoon	18 g	2 full tablespoon	32 g	4 full tablespoon	36 g
Mozzarella cheese	1 slice	20 g	1 and ½ slice	30 g	2 slices	40 g	3 slices	60 g
Ricotta cheese	1 small slice	15 g	1 medium slice	35 g	1 large slice	45 g	2 large slices	90 g
Muenster cheese	1 small slice	25 g	1 medium slice	35 g	1 large slice	50 g	2 medium large slices	70 g
Sour cultured, Cream half-half	1 teaspoon	10 g	1 level tablespoon	15 g	1 full tablespoon	25 g	4 level tablespoon	60 g
American cheese	1 level dessert spoon	10 g	1 level tablespoon	15 g	1 full tablespoon	30 g	2 full tablespoon	60 g
Yogurt, plan	½ pot	100 g	1 pot	200 g	1 and ½ pots	300 g	2 pots	400 g
Yogurt, fruit	1 pot	100 g	1 and ½ pots	150 g	2 pots	200 g	3 pots	300 g
**Oils and fats**								
Margarine	1 level teaspoon	4 g	1 full teaspoon	8 g	1 level dessert spoon	13 g	1 full dessert spoon	23 g
Butter	1 level teaspoon	4 g	1 full teaspoon	8 g	1 level dessert spoon	13 g	1 full dessert spoon	23 g
Mayonnaise	1 full teaspoon	6 g	2 full teaspoon	12 g	1 full dessert spoon	17 g	2 full dessert spoon	34 g
Goose pate	1 full teaspoon	8 g	2 full teaspoon	16 g	1 full dessert spoon	21 g	3 full dessert spoon	63 g
Oil, add	1 teaspoon	2 ml	2 teaspoon	4 ml	1 dessert spoon	5 ml	1 tablespoon	8 ml
**Sugars and sweets**								
Sago	3 full tablespoon	90 g	4 full tablespoon	120 g	5 full tablespoon	150 g	6 full tablespoon	180 g
Chocolate	2 pieces	15 g	3 pieces	30 g	4 pieces	40 g	8 pieces	80 g
Flan, pudding	1 full tablespoon	50 g	2 full tablespoon	90 g	3 full tablespoon	130 g	5 full tablespoon	220 g
Ice cream	1 full tablespoon	55 g	1 ball	75 g	1 cup	100 g	2 balls	150 g
Gelatin	2 full tablespoon	50 g	3 full tablespoon	75 g	5 full tablespoon	125 g	12 full tablespoon	300 g
Condensed milk	1 level teaspoon	10 g	1 level dessert spoon	15 g	1 full tablespoon	40 g	2 full dessert spoon	50 g
Jelly	1 full teaspoon	10 g	2 full teaspoon	20 g	1 full tablespoon	34 g	2 full tablespoon	68 g
Honey	1 dessert spoon	10 g	1 tablespoon	15 g	2 dessert spoon	20 g	2 tablespoon	30 g
Chocolate, dry	1 level dessert spoon	7 g	1 level tablespoon	11 g	1 full tablespoon	16 g	2 full tablespoon	32 g
**Beverages**								
Coffee, brewed	¼ cup	50 ml	½ cup	100 ml	¾ cup	150 ml	1 cup	200 ml
Coffee, instant	1 teaspoon	1.5 g	2 teaspoon	3 g	4 teaspoon	6 g	6 teaspoon	9 g
Tea	¾ cup	150 ml	1 cup	200 ml	1 and ¼ cups	250 ml	1 mug	300 ml
Soft drink	1 cup	200 ml	1 full glass	250 ml	1 can	350 ml	2 full glass	500 ml
Fruit juice raw	¾ cup	150 ml	1 cup	200 ml	1 full glass	250 ml	2 cups	400 ml
Fruit juice artificial	¾ cup	150 ml	1 cup	200 ml	1 full glass	250 ml	2 full glass	500 ml
Soymilk	¾ cup	150 ml	½ glass	175 ml	1 cup	200 ml	1 full glass	250 ml
Beer	1 glass	300 ml	1 bottle	600 ml	1 and ½ bottles	900 ml	6 bottles	3600 ml
Wine	½ glass	75 ml	¾ glass	115 ml	1 glass	150 ml	2 glass	300 ml

The FFQ also included open questions about frequency of food consumption and eight queries about food preferences and usual dietary practices: number of meals per day, type of sweetener added in beverages, type and amount of fat used in food preparation, if intake of visible fat from meats, the habit of salt added in prepared foods and salads, and other foods and/or seasonings not listed but regularly consumed.

## Discussion

Patients with diabetes are encouraged to comply with specific dietary recommendations to achieve optimal glucose, lipid, and blood pressure control as well as a healthy body weight [2]. These aspects can modify the food intake of patients with diabetes as compared to the general population. We constructed a quantitative FFQ and a portfolio with photos of 98 food items distributed into nine food groups and based on WDR performed by patients with type 2 diabetes. This is the first FFQ for Brazilian type 2 diabetes patients.

The development of an FFQ should take into account some important aspects such as drawing up the food list, definition of portion intake [8], and how representative of the dietary habits of a population-based sample is the food list [1]. Our FFQ took into account the foods most commonly consumed by patients with type 2 diabetes and, as recommended, represents the regional dietary habits [1] in Southern Brazil. In addition, the cultural and clinical appropriateness of food items included in our FFQ was assured by using as reference the 3-day WDRs, a dietary instrument previously standardized, validated [12,27], and widely used in diabetic patients by our research group [11,28-30]. It is also important to keep in mind that these WDR were performed throughout the year because it is known that portion sizes and food types can vary according to season [31] and the gender distribution was equal, since gender also influences food intake [31].

The final food list was drawn up considering the contribution criteria of each relevant nutrient to minimize the omission of usually consumed food [14]. It should be noted that nutrients known to influence glucose, lipid, or blood pressure control, or that have been associated with chronic diabetic complications were considered to choose the relevant nutrients for the food list. The number of food items in the final version of the FFQ is appropriate according to suggestions found in the literature [32] and similar to other FFQs for diabetes around the world [5-7]. Small food lists (less than 50 items) may underestimate food intake, and very long lists (more than 100 items) may tire respondents and overestimate food intake [32].

The FFQ in the present study also includes a quantitative evaluation of food intake. The size of portions (quartiles of intake) was based on the weight of consumed foods assessed by 3-day WDR. These portions, specific for each food item, were shown as photos and as household measures in the food portfolio and can be easily used for respondents to select their own portion size [8]. Finally, the FFQ structure including open questions provides greater freedom to choose the actual frequency of food intake and reduces the error of consumption categories by the patients [32]. The frequency of food consumption was considered in this FFQ (day, week, month, or year). However, care should be taken when assessing the consumption of a particular food per year. The diary conversion of intake is necessary to minimize the contribution of the foods scarcely consumed in evaluating the eating habits of the individual [8].

## Conclusions

In conclusion, we developed a practical quantitative FFQ and a portfolio with 98 food items covering the past 12 months and representing the usual food intake of patients with type 2 diabetes in Southern Brazil. This relatively long-term evaluation of food intake can be particularly relevant for prospective studies that evaluate associations of diet with chronic diabetic complications. However, this dietary instrument should be validated in other samples of patients.

## Competing interests

The authors declare that they have no competing interests.

## Authors’ contributions

RAS was responsible for the study design, collection, analysis and interpretation of data, as well as the preparation of the manuscript. BPR contributed the construction of the portfolio with food photos. TCR contributed the initial idea. MJA contributed to the interpretation of results and final version of the manuscript. JCA contributed to the study design and each step of the FFQ construction as well as proof-reading the manuscript. The final paper version was approved by all authors.

## Pre-publication history

The pre-publication history for this paper can be accessed here:

http://www.biomedcentral.com/1471-2458/13/740/prepub

## Supplementary Material

Additional file 1 Food frequency questionnaire developed for Brazilian patients with type 2 Diabetes. Click here for file
