# Supplementary material for: Development of a quantitative food frequency questionnaire for Brazilian patients with type 2 diabetes
Source: BMC Public Health. 2013 Aug 9;13:740. doi: 10.1186/1471-2458-13-740 (PMC3751547; doi:10.1186/1471-2458-13-740)
Supplement: Additional file 1 — Food frequency questionnaire developed for Brazilian patients with type 2 Diabetes. [file 1471-2458-13-740-S1.doc]

| **FOOD** | **HOW OFTEN DO YOU EAT** | | | | **PORTION** | |
| --- | --- | --- | --- | --- | --- | --- |
| **CEREALS, TUBERS, ROOTS, AND DERIVATIVES** | | | | | | |
| White rice | N 1 2 3 4 5 6 7 8 9 10 11 12 | | D W M Y | | ÿ 2 full tablespoon  ÿ 4 full tablespoon  ÿ 5 full tablespoon  ÿ 8 full tablespoon | 50 g  100 g  125 g  200 g |
| Spaghetti pasta | N 1 2 3 4 5 6 7 8 9 10 11 12 | | D W M Y | | ÿ 3 full tablespoon  ÿ 4 full tablespoon  ÿ 1 paten  ÿ 1 full paten | 75 g  100 g  200 g  320 g |
| Cassava, boiled/fried | N 1 2 3 4 5 6 7 8 9 10 11 12 | | D W M Y | | ÿ 2 pieces  ÿ 3 pieces  ÿ 4 pieces  ÿ 6 pieces | 60 g  90 g  120 g  240 g |
| Potato, boiled/roasted/fried | N 1 2 3 4 5 6 7 8 9 10 11 12 | | D W M Y | | ÿ 2 full tablespoon  ÿ 3 full tablespoon  ÿ 4 full tablespoon  ÿ 6 full tablespoon | 60 g  90 g  120 g  180 g |
| Maize porridge, boiled/fried | N 1 2 3 4 5 6 7 8 9 10 11 12 | | D W M Y | | ÿ 1 serving spoon  ÿ 2 full tablespoon  ÿ 4 full tablespoon  ÿ 1 paten | 60 g  90 g  150 g  325 g |
| French or Vienna bread | N 1 2 3 4 5 6 7 8 9 10 11 12 | | D W M Y | | ÿ ½ unit  ÿ 1 unit  ÿ 1 ½ units  ÿ 2 units | 25 g  50 g  75 g  100 g |
| White bread | N 1 2 3 4 5 6 7 8 9 10 11 12 | | D W M Y | | ÿ 1 slice  ÿ 2 slices  ÿ 2 ½ slices  ÿ 3 ½ slices | 25 g  50 g  62,5 g  87,5 g |
| Whole bread | N 1 2 3 4 5 6 7 8 9 10 11 12 | | D W M Y | | ÿ ½ slice  ÿ 1 slice  ÿ 2 slices  ÿ 3 slices | 15 g  30 g  60 g  90 g |
| Homemade bread | N 1 2 3 4 5 6 7 8 9 10 11 12 | | D W M Y | | ÿ 2/3 slice  ÿ 1 slice  ÿ 1 ½ slices  ÿ 2 ½ slices | 60 g  68 g  86 g  145 g |
| Cake | N 1 2 3 4 5 6 7 8 9 10 11 12 | | D W M Y | | ÿ 1 small slice  ÿ 1 medium slice  ÿ 1 large slice  ÿ 2 medium slices | 50 g  70 g  90 g  140 g |
| Wheat cracker | N 1 2 3 4 5 6 7 8 9 10 11 12 | | D W M Y | | ÿ 4 units  ÿ 6 units  ÿ 9 units  ÿ 20 units | 20 g  30 g  45 g  100 g |
| Milk cracker | N 1 2 3 4 5 6 7 8 9 10 11 12 | | D W M Y | | ÿ 5 units  ÿ 8 units  ÿ 11 units  ÿ 32 units | 25 g  40 g  55 g  160 g |
| **List other foods, seasoning or preparations of this group that do you usually eat or drink and not was mentioned:** | | | | | | |
| FOOD | | FREQUENCY | | AMOUNT CONSUMED | | |
|  | |  | |  | | |
|  | |  | |  | | |
|  | |  | |  | | |
| **VEGETABLES AND LEGUMES** | | | | | | |
| Carrot | N 1 2 3 4 5 6 7 8 9 10 11 12 | | D W M Y | | ÿ 2 full tablespoon  ÿ 3 full tablespoon  ÿ 5 full tablespoon  ÿ 10 full tablespoon | 24 g  36 g  60 g  120 g |
| Tomato | N 1 2 3 4 5 6 7 8 9 10 11 12 | | D W M Y | | ÿ 3 small slices  ÿ 5 small slices  ÿ 7 small slices  ÿ 7 medium slices | 30 g  50 g  70 g  100 g |
| Chayote | N 1 2 3 4 5 6 7 8 9 10 11 12 | | D W M Y | | ÿ 1 full tablespoon  ÿ 2 full tablespoon  ÿ 3 ½ full tablespoon  ÿ 5 full tablespoon | 30 g  60 g  100 g  145 g |
| Cabbage | N 1 2 3 4 5 6 7 8 9 10 11 12 | | D W M Y | | ÿ 4 full tablespoon  ÿ 7 full tablespoon  ÿ 10 full tablespoon  ÿ 6 full medium skimmer | 40 g  70 g  100 g  150 g |
| Lettuce | N 1 2 3 4 5 6 7 8 9 10 11 12 | | D W M Y | | ÿ 1 tagger  ÿ 2 taggers  ÿ 5 medium leaf  ÿ 1 full paten | 20 g  30 g  50 g  80 g |
| Watercress | N 1 2 3 4 5 6 7 8 9 10 11 12 | | D W M Y | | ÿ 1 full dessert plate  ÿ 2 taggers  ÿ 1 full paten  ÿ 2 full paten | 20 g  30 g  80 g  160 g |
| Kale, spinach | N 1 2 3 4 5 6 7 8 9 10 11 12 | | D W M Y | | ÿ 2 full tablespoon  ÿ 3 full tablespoon  ÿ 5 full tablespoon  ÿ 9 full tablespoon | 40 g  60 g  100 g  180 g |
| Broccoli, cauliflower | N 1 2 3 4 5 6 7 8 9 10 11 12 | | D W M Y | | ÿ 1 small bunch  ÿ 1 medium bunch  ÿ 1 large bunch  ÿ 2 medium bunches | 30 g  60 g  100 g  130 g |
| Snap bean | N 1 2 3 4 5 6 7 8 9 10 11 12 | | D W M Y | | ÿ 2 level tablespoon  ÿ 2 full tablespoon  ÿ 5 full tablespoon  ÿ 15 full tablespoon | 30 g  40 g  100 g  300 g |
| Pumpkin | N 1 2 3 4 5 6 7 8 9 10 11 12 | | D W M Y | | ÿ 1 medium piece  ÿ 2 medium pieces  ÿ 2 ½ medium pieces  ÿ 6 medium pieces | 50 g  100 g  125 g  300 g |
| Beet | N 1 2 3 4 5 6 7 8 9 10 11 12 | | D W M Y | | ÿ 2 medium slices  ÿ 5 medium slices  ÿ 8 medium slices  ÿ 12 medium slices | 30 g  60 g  90 g  140 g |
| **List other foods, seasoning or preparations of this group that do you usually eat or drink and not was mentioned:** | | | | | | |
| FOOD | | FREQUENCY | | AMOUNT CONSUMED | | |
|  | |  | |  | | |
|  | |  | |  | | |
|  | |  | |  | | |
| **FRUITS** | | | | | | |
| Banana | N 1 2 3 4 5 6 7 8 9 10 11 12 | | D W M Y | | ÿ 1 small unit  ÿ 1 medium unit  ÿ 1 large unit  ÿ 2 medium units | 40 g  70 g  90 g  140 g |
| Apple, pear | N 1 2 3 4 5 6 7 8 9 10 11 12 | | D W M Y | | ÿ 1 small unit  ÿ 1 ½ small units  ÿ 1 medium unit  ÿ 1 large unit | 90 g  135 g  150 g  230 g |
| Orange, tangerine | N 1 2 3 4 5 6 7 8 9 10 11 12 | | D W M Y | | ÿ 1 small unit  ÿ 1 ½ small unit  ÿ 1 large unit  ÿ 2 medium units | 90 g  135 g  180 g  225 g |
| Papaya | N 1 2 3 4 5 6 7 8 9 10 11 12 | | D W M Y | | ÿ ½ small slice  ÿ 1 small slice  ÿ ¼ unit  ÿ ½ unit | 80 g  100 g  135 g  270 g |
| Mango | N 1 2 3 4 5 6 7 8 9 10 11 12 | | D W M Y | | ÿ 1 small piece  ÿ 2 small pieces  ÿ 1 medium piece  ÿ 6 small pieces | 60 g  120 g  140 g  360 g |
| Grape | N 1 2 3 4 5 6 7 8 9 10 11 12 | | D W M Y | | ÿ 8 units  ÿ 14 units  ÿ 1 small bunch  ÿ 1 medium bunch | 64 g  112 g  170 g  350 g |
| Persimmon | N 1 2 3 4 5 6 7 8 9 10 11 12 | | D W M Y | | ÿ 1 small unit  ÿ 1 large unit  ÿ 2 medium units  ÿ 3 small units | 85 g  150 g  220 g  255 g |
| Casaba melon | N 1 2 3 4 5 6 7 8 9 10 11 12 | | D W M Y | | ÿ ½ small slice  ÿ 1 small slice  ÿ 1 medium slice  ÿ 1 large slice | 78 g  125 g  200 g  300 g |
| Watermelon | N 1 2 3 4 5 6 7 8 9 10 11 12 | | D W M Y | | ÿ 1 small slice  ÿ 1 medium slice  ÿ 1 large slice  ÿ 2 medium slices | 143 g  200 g  282,5 g  350 g |
| **List other foods, seasoning or preparations of this group that do you usually eat or drink and not was mentioned:** | | | | | | |
| FOOD | | FREQUENCY | | AMOUNT CONSUMED | | |
|  | |  | |  | | |
|  | |  | |  | | |
|  | |  | |  | | |
| **MEAT AND EGGS** | | | | | | |
| Beef, boiled/roasted/fried | N 1 2 3 4 5 6 7 8 9 10 11 12 | | D W M Y | | ÿ 1 small slice  ÿ 4 small pieces  ÿ 1 large slice  ÿ 2 large slices | 70 g  80 g  135 g  270 g |
| Ground beef | N 1 2 3 4 5 6 7 8 9 10 11 12 | | D W M Y | | ÿ 2 full tablespoon  ÿ 3 full tablespoon  ÿ 4 full tablespoon  ÿ 8 full tablespoon | 50 g  75 g  100 g  200 g |
| Beef steak | N 1 2 3 4 5 6 7 8 9 10 11 12 | | D W M Y | | ÿ ½ small unit  ÿ 1 small unit  ÿ 1 medium unit  ÿ 2 medium units | 40 g  80 g  100 g  200 g |
| Beef liver | N 1 2 3 4 5 6 7 8 9 10 11 12 | | D W M Y | | ÿ ½ large unit  ÿ 1 small unit  ÿ 1 medium unit  ÿ 1 large unit | 75 g  80 g  100 g  150 g |
| Chicken thigh, boiled/roasted/fried | N 1 2 3 4 5 6 7 8 9 10 11 12 | | D W M Y | | ÿ 1 medium piece  ÿ 1 large piece  ÿ 2 medium pieces  ÿ 3 medium pieces | 60 g  95 g  110 g  180 g |
| Chicken breast, boiled/roasted/fried | N 1 2 3 4 5 6 7 8 9 10 11 12 | | D W M Y | | ÿ 1 medium piece  ÿ 1 large piece  ÿ 2 medium pieces  ÿ 3 medium pieces | 60 g  95 g  110 g  180 g |
| Fish, boiled/roasted/fried | N 1 2 3 4 5 6 7 8 9 10 11 12 | | D W M Y | | ÿ ½ small piece  ÿ 1 small piece  ÿ 1 large piece  ÿ 2 large pieces | 60 g  100 g  155 g  310 g |
| Pork, boiled/roasted/fried | N 1 2 3 4 5 6 7 8 9 10 11 12 | | D W M Y | | ÿ 1 small slice  ÿ 1 medium slice  ÿ 1 large slice  ÿ 2 medium slices | 60 g  90 g  120 g  180 g |
| Luncheon/bologna | N 1 2 3 4 5 6 7 8 9 10 11 12 | | D W M Y | | ÿ ½ unit  ÿ 1 unit  ÿ 1 ½ units  ÿ 2 ½ units | 30 g  60 g  90 g  150 g |
| Frankfurter wiener, hot dog | N 1 2 3 4 5 6 7 8 9 10 11 12 | | D W M Y | | ÿ 1 unit  ÿ 1 ½ units  ÿ 2 units  ÿ 3 ½ units | 42 g  63 g  84 g  147 g |
| Mortadella, ham, salami | N 1 2 3 4 5 6 7 8 9 10 11 12 | | D W M Y | | ÿ 1 medium slice  ÿ 1 large slice  ÿ 2 medium slices  ÿ 2 large slices | 15 g  25 g  30 g  50 g |
| Egg, boiled/fried | N 1 2 3 4 5 6 7 8 9 10 11 12 | | D W M Y | | ÿ ½ unit  ÿ 1 unit  ÿ 1 ½ units  ÿ 3 units | 25 g  50 g  75 g  150 g |
| **List other foods, seasoning or preparations of this group that do you usually eat or drink and not was mentioned:** | | | | | | |
| FOOD | | FREQUENCY | | AMOUNT CONSUMED | | |
|  | |  | |  | | |
|  | |  | |  | | |
|  | |  | |  | | |
| **OILS AND FATS** | | | | | | |
| Margarine | N 1 2 3 4 5 6 7 8 9 10 11 12 | | D W M Y | | ÿ 1 level teaspoon  ÿ 1 full teaspoon  ÿ 1 level dessert spoon  ÿ 1 full dessert spoon | 4 g  8 g  13 g  23 g |
| Butter | N 1 2 3 4 5 6 7 8 9 10 11 12 | | D W M Y | | ÿ 1 level teaspoon  ÿ 1 full teaspoon  ÿ 1 level dessert spoon  ÿ 1 full dessert spoon | 4 g  8 g  13 g  23 g |
| Mayonnaise | N 1 2 3 4 5 6 7 8 9 10 11 12 | | D W M Y | | ÿ 1 full teaspoon  ÿ 2 full teaspoon  ÿ 1 full dessert spoon  ÿ 2 full dessert spoon | 6 g  12 g  17 g  34 g |
| Goose pate | N 1 2 3 4 5 6 7 8 9 10 11 12 | | D W M Y | | ÿ 1 full teaspoon  ÿ 2 full teaspoon  ÿ 1 full dessert spoon  ÿ 3 full dessert spoon | 8 g  16 g  21 g  63 g |
| Oil, add | N 1 2 3 4 5 6 7 8 9 10 11 12 | | D W M Y | | ÿ 1 teaspoon  ÿ 2 teaspoon  ÿ 1 dessert spoon  ÿ 1 tablespoon | 2 ml  4 ml  5 ml  8 ml |
| **List other foods, seasoning or preparations of this group that do you usually eat or drink and not was mentioned:** | | | | | | |
| FOOD | | FREQUENCY | | AMOUNT CONSUMED | | |
|  | |  | |  | | |
|  | |  | |  | | |
|  | |  | |  | | |
| **BEANS** | | | | | | |
| Beans (all types) | N 1 2 3 4 5 6 7 8 9 10 11 12 | | D W M Y | | ÿ 1 small full scoop  ÿ 1 level medium scoop  ÿ 2 small full scoop  ÿ 2 level medium scoop | 65 g  80 g  130 g  160 g |
| Lentil | N 1 2 3 4 5 6 7 8 9 10 11 12 | | D W M Y | | ÿ 1 level medium scoop  ÿ 1 medium full scoop  ÿ 2 level medium scoop  ÿ 2 medium full scoop | 100 g  160 g  200 g  320 g |
| **List other foods, seasoning or preparations of this group that do you usually eat or drink and not was mentioned:** | | | | | | |
| FOOD | | FREQUENCY | | AMOUNT CONSUMED | | |
|  | |  | |  | | |
|  | |  | |  | | |
|  | |  | |  | | |
| **MILK AND DAIRY PRODUCTS** | | | | | | |
| Milk, fluid, 3.25% fat | N 1 2 3 4 5 6 7 8 9 10 11 12 | | D W M Y | | ÿ ½ cup  ÿ ¾ cup  ÿ 1 cup  ÿ 1 mug | 100 ml  150 ml  200 ml  300 ml |
| Milk, fluid, 2% fat | N 1 2 3 4 5 6 7 8 9 10 11 12 | | D W M Y | | ÿ ½ cup  ÿ ¾ cup  ÿ 1 cup  ÿ 1 mug | 100 ml  150 ml  200 ml  300 ml |
| Milk, fluid, nonfat | N 1 2 3 4 5 6 7 8 9 10 11 12 | | D W M Y | | ÿ ¾ cup  ÿ 1 cup  ÿ 1 glass  ÿ 1 ¼ cups | 150 ml  200 ml  240 ml  250 ml |
| Milk, dry | N 1 2 3 4 5 6 7 8 9 10 11 12 | | D W M Y | | ÿ 1 full tablespoon  ÿ 2 full dessert spoon  ÿ 2 full tablespoon  ÿ 4 full dessert spoon | 16 g  18 g  32 g  36 g |
| Mozzarella cheese | N 1 2 3 4 5 6 7 8 9 10 11 12 | | D W M Y | | ÿ 1 slice  ÿ 1 ½ slices  ÿ 2 slices  ÿ 3 slices | 20 g  30 g  40 g  60 g |
| Ricotta cheese | N 1 2 3 4 5 6 7 8 9 10 11 12 | | D W M Y | | ÿ 1 small slice  ÿ 1 medium slice  ÿ 1 large slice  ÿ 2 large slices | 15 g  35 g  45 g  90 g |
| Muenster cheese | N 1 2 3 4 5 6 7 8 9 10 11 12 | | D W M Y | | ÿ 1 small slice  ÿ 1 medium slice  ÿ 1 large slice  ÿ 2 medium slices | 25 g  35 g  50 g  70 g |
| Sour cultured, Cream half-half | N 1 2 3 4 5 6 7 8 9 10 11 12 | | D W M Y | | ÿ 1 level dessert spoon  ÿ 1 level tablespoon  ÿ 1 full tablespoon  ÿ 2 full tablespoon | 10 g  15 g  30 g  60 g |
| American cheese | N 1 2 3 4 5 6 7 8 9 10 11 12 | | D W M Y | | ÿ 1 teaspoon  ÿ 1 level tablespoon  ÿ 1 full tablespoon  ÿ 4 level tablespoon | 10 g  15 g  25 g  60 g |
| Yogurt, plan | N 1 2 3 4 5 6 7 8 9 10 11 12 | | D W M Y | | ÿ ½ pot  ÿ 1 pot  ÿ 1 ½ pots  ÿ 2 pots | 100 g  200 g  300 g  400 g |
| Yogurt, fruit | N 1 2 3 4 5 6 7 8 9 10 11 12 | | D W M Y | | ÿ1 pot  ÿ 1 ½ pot  ÿ 2 pots  ÿ 3 pots | 100 g  150 g  200 g  300 g |
| **List other foods, seasoning or preparations of this group that do you usually eat or drink and not was mentioned:** | | | | | | |
| FOOD | | FREQUENCY | | AMOUNT CONSUMED | | |
|  | |  | |  | | |
|  | |  | |  | | |
|  | |  | |  | | |
| **BEVERAGES** | | | | | | |
| Coffee, brewed | N 1 2 3 4 5 6 7 8 9 10 11 12 | | D W M Y | | ÿ ¼ cup  ÿ ½ cup  ÿ ¾ cup  ÿ 1 cup | 50 ml  100 ml  150 ml  200 ml |
| Coffee, instant | N 1 2 3 4 5 6 7 8 9 10 11 12 | | D W M Y | | ÿ 1 teaspoon  ÿ 2 teaspoon  ÿ 4 teaspoon  ÿ 6 teaspoon | 1,5 g  3 g  6 g  9 g |
| Tea | N 1 2 3 4 5 6 7 8 9 10 11 12 | | D W M Y | | ÿ ¾ cup  ÿ 1 cup  ÿ 1 ¼ cups  ÿ 1 mug | 150 ml  200 ml  250 ml  300 ml |
| Soft drink | N 1 2 3 4 5 6 7 8 9 10 11 12 | | D W M Y | | ÿ 1 cup  ÿ 1 full glass  ÿ 1 can  ÿ 2 full glass | 200 ml  250 ml  350 ml  500 ml |
| Fruit juice raw | N 1 2 3 4 5 6 7 8 9 10 11 12 | | D W M Y | | ÿ ¾ cup  ÿ 1 cup  ÿ 1 full glass  ÿ 2 cups | 150 ml  200 ml  250 ml  400 ml |
| Fruit juice artificial | N 1 2 3 4 5 6 7 8 9 10 11 12 | | D W M Y | | ÿ ¾ cup  ÿ 1 cup  ÿ 1 full glass  ÿ 2 full glass | 150 ml  200 ml  250 ml  500 ml |
| Soymilk | N 1 2 3 4 5 6 7 8 9 10 11 12 | | D W M Y | | ÿ ¾ cup  ÿ ½ glass  ÿ 1 cup  ÿ 1 full glass | 150 ml  175 ml  200 ml  250 ml |
| Beer | N 1 2 3 4 5 6 7 8 9 10 11 12 | | D W M Y | | ÿ 1 glass  ÿ 1 bottle  ÿ 1 ½ bottles  ÿ 6 bottles | 300 ml  600 ml  900 ml  3600 ml |
| Wine | N 1 2 3 4 5 6 7 8 9 10 11 12 | | D W M Y | | ÿ ½ glass  ÿ ¾ glass  ÿ 1 glass  ÿ 2 glass | 75 ml  115 ml  150 ml  300 ml |
| **List other foods, seasoning or preparations of this group that do you usually eat or drink and not was mentioned:** | | | | | | |
| FOOD | | FREQUENCY | | AMOUNT CONSUMED | | |
|  | |  | |  | | |
|  | |  | |  | | |
|  | |  | |  | | |
| **SUGARS AND SWEETS** | | | | | | |
| Sago | N 1 2 3 4 5 6 7 8 9 10 11 12 | | D W M Y | | ÿ 3 full tablespoon  ÿ 4 full tablespoon  ÿ 5 full tablespoon  ÿ 6 full tablespoon | 90 g  120 g  150 g  180 g |
| Chocolate | N 1 2 3 4 5 6 7 8 9 10 11 12 | | D W M Y | | ÿ 2 pieces  ÿ 3 pieces  ÿ 4 pieces  ÿ 8 pieces | 15 g  30 g  40 g  80 g |
| Flan, pudding | N 1 2 3 4 5 6 7 8 9 10 11 12 | | D W M Y | | ÿ 1 full tablespoon  ÿ 2 full tablespoon  ÿ 3 full tablespoon  ÿ 5 full tablespoon | 50 g  90 g  130 g  220 g |
| Ice cream | N 1 2 3 4 5 6 7 8 9 10 11 12 | | D W M Y | | ÿ 1 full tablespoon  ÿ 1 ball  ÿ 1 cup  ÿ 2 balls | 55 g  75 g  100 g  150 G |
| Gelatin | N 1 2 3 4 5 6 7 8 9 10 11 12 | | D W M Y | | ÿ 2 full tablespoon  ÿ 3 full tablespoon  ÿ 5 full tablespoon  ÿ 12 full tablespoon | 50 g  75 g  125 g  300 g |
| Condensed milk | N 1 2 3 4 5 6 7 8 9 10 11 12 | | D W M Y | | ÿ 1 level teaspoon  ÿ 1 level dessert spoon  ÿ 1 full tablespoon  ÿ 2 full dessert spoon | 10 g  15 g  40 g  50 g |
| Jelly | N 1 2 3 4 5 6 7 8 9 10 11 12 | | D W M Y | | ÿ 1 full teaspoon  ÿ 2 full teaspoon  ÿ 1 full tablespoon  ÿ 2 full tablespoon | 10 g  20 g  34 g  68 g |
| Honey | N 1 2 3 4 5 6 7 8 9 10 11 12 | | D W M Y | | ÿ 1 dessert spoon  ÿ 1 tablespoon  ÿ 2 dessert spoon  ÿ 2 tablespoon | 10 g  15 g  20 g  30 g |
| Chocolate, dry | N 1 2 3 4 5 6 7 8 9 10 11 12 | | D W M Y | | ÿ 1 level dessert spoon  ÿ 1 level tablespoon  ÿ 1 full tablespoon  ÿ 2 full tablespoon | 7 g  11 g  16 g  32 g |
| **List other foods, seasoning or preparations of this group that do you usually eat or drink and not was mentioned:** | | | | | | |
| FOOD | | FREQUENCY | | AMOUNT CONSUMED | | |
|  | |  | |  | | |
|  | |  | |  | | |
|  | |  | |  | | |

**LEGEND:**

**D = day N = never W = week M = month Y = year**
